# Evaluation of the Utility of Hybrid PET/MR Neuroimaging in Inflammatory Demyelination and Encephalitis

**DOI:** 10.3390/jcm14082736

**Published:** 2025-04-16

**Authors:** Radosław Zawadzki, Maciej Naumowicz, Magdalena Zalewska, Joanna Zajkowska, Bożena Kubas

**Affiliations:** 1 Department of Radiology, Medical University of Białystok, M. Skłodowskiej-Curie 24A, 15-276 Białystok, Poland; zaw.radoslaw@gmail.com (R.Z.); 41519@student.umb.edu.pl (M.Z.); bozena.kubas@umb.edu.pl (B.K.); 2 Department of Infectious Diseases and Neuroinfections, Medical University of Białystok, Żurawia 14, 15-540 Białystok, Poland; joanna.zajkowska@umb.edu.pl

**Keywords:** PET/MRI, multiple sclerosis, inflammatory demyelination, AIE, encephalitis, neuroimaging

## Abstract

With the increased availability of hybrid PET/MRI in recent years, this method is increasingly used for neuroimaging in clinical practice. It combines the advantages of MRI (including high-resolution imaging of intracerebral lesions and data provided from specialised MRI sequences) with the benefits of PET, which visualises functional alterations in the brain, as well as assesses the myelin quantity changes and the severity of inflammation. The use of PET/MRI may help to eliminate the limitations of MRI indicated in imaging demyelinating inflammatory diseases (such as low specificity in imaging demyelination and a weak correlation of findings with clinical symptoms), as well as insufficient sensitivity in detecting lesions present in encephalitis. In addition to supporting the diagnosis of encephalitis, PET/MRI facilitates monitoring of the disease course and assessing the treatment efficacy of inflammatory demyelinating diseases and encephalitis, as well as evaluating the risk of multiple sclerosis relapse. Further multi-centre longitudinal studies are necessary to assess the real clinical potential of PET/MRI among patients with inflammatory demyelination or encephalitis. In addition to MS and AIE, these studies should also include other inflammatory demyelinating diseases (ADEM, NMO, NMOSD, and MOGAD) as well as encephalitis of viral and parasitic aetiology.

## 1. Introduction

Diagnosing encephalitis, particularly seronegative autoimmune encephalitis (AIE), presents several challenges. It is not uncommon for patients to have a normal magnetic resonance imaging (MRI) scan [1]. Moreover, MRI is not a perfect imaging method for assessing inflammatory demyelination. It has several weaknesses, including low specificity in identifying the severity of demyelination and the activity of the inflammatory process. Furthermore, there is a poor correlation between the imaged lesions and clinical symptoms presented by the patient. Considering the above-mentioned imperfections of MRI technology, we suggest that the use of a hybrid PET/MRI (positron emission tomography/magnetic resonance imaging) system could help eliminate these limitations and bring potential benefits to patients and clinicians [1,2,3]. This system allows for to combination of the advantages of PET (i.e., the high specificity in imaging areas of demyelination, the possibility of assessing the severity of neuroinflammation, as well as detecting areas of hypo- and hypermetabolism) with the high resolution of MRI. The purpose of our paper is to evaluate the usefulness of hybrid PET/MRI in inflammatory demyelination and encephalitis in aspects of the diagnosis and differential diagnosis of these diseases, exploring their aetiopathogenesis, correlating imaging results with clinical symptoms, assessing the course of the disease and its prognosis, as well as evaluating the effectiveness of the applied treatment.

## 2. Materials and Methods

The PubMed, Web of Science and Scopus databases were searched using the combination (1) + (2) of the following keywords: (1): ‘PET-MRI’ or ‘PET/MRI’; (2): ‘encephalitis’, ‘autoimmune encephalitis’, ‘parasitic encephalitis’, ‘viral encephalitis’, ‘inflammatory demyelination’, ‘multiple sclerosis’, ‘ADEM’, ‘NMO’, ‘NMOSD’, ‘MOGAD’. Our review includes studies published between 2004 and 2024 (until 1 October 2024). Papers that have not been published in English were excluded. We have included relevant articles in order to assess the utility of hybrid PET/MR imaging among patients with inflammatory demyelination and encephalitis as accurately as possible. The included papers were original research studies or clinical case reports that used hybrid PET/MRI or specialised software combining PET and MRI scans acquired from patients with inflammatory demyelination or encephalitis of autoimmune, viral, or parasitic aetiology. We analysed these articles in terms of the diagnostic superiority of PET/MRI over other imaging modalities, its utility in monitoring the course of the disease, its ability to correlate clinical symptoms with imaged lesions, its assessment of treatment efficacy, and its evaluation of prognoses among patients with the mentioned diseases. We included data on the following: comparison of the sensitivity and specificity of PET/MRI in the diagnosis of inflammatory demyelination and encephalitis with the sensitivity and specificity of other imaging modalities (MRI, PET, and CT separately); correlation of the results from clinical severity scales with tracer uptake levels in various brain areas; descriptions of the correlation of PET/MRI findings with patient prognosis; correlations between tracer uptake in different brain locations with lesions detected with MRI; as well as correlations between imaging results obtained with different tracers used on the same patients.

## 3. Summary of MRI Techniques and Sequences Useful in Neuroimaging of Inflammatory Demyelination and Encephalitis

The basic modalities used in MRI are T1-weighted (T1-w) and T2-weighted (T2-w) imaging, which rely on the differences in tissue relaxation times (T1 and T2). They are valuable for assessing anatomical structures and identifying pathological lesions, including demyelinating lesions and neuroinflammation [4,5].

The Fluid-Attenuated Inversion Recovery (FLAIR) sequence is a modification of the T2-weighted sequence that eliminates the signal from cerebrospinal fluid, which appears dark, thus allowing better visualisation of lesions near the fissures or in the periventricular areas [6].

SPACE (sampling perfection with application-optimised contrasts using different flip-angle evolutions) is a 3D turbo spin-echo (TSE) MRI sequence developed by Siemens that is used for vascular wall imaging because of its high sampling efficiency and good blood suppression properties. SPACE enhances the visualisation of spinal cord inflammation by reducing cerebrospinal fluid flow artefacts and improving the contrast between spinal cord tissue and lesions [7,8].

MPRAGE (Magnetisation Prepared Rapid Gradient Echo) provides good spatial resolution, a high signal-to-noise ratio, and good contrast between grey and white matter. It effectively visualises the boundaries of lesions occurring during demyelination [9].

Diffusion-weighted imaging (DWI) visualises the movement of water protons in tissue, enabling the creation of diffusion and apparent diffusion coefficient (ADC) maps [10].

Diffusion tensor imaging (DTI) is a more precise method of evaluating the diffusion in different directions in the tissue, which allows the course of axonal fibres to be visualised [11]. A number of parameters are analysed in DTI. Fractional anisotropy (FA) is considered a quantitative indicator of white matter integrity, although it is not specific to myelin [12]. Mean diffusivity (MD) characterises the average diffusivity of water and is sensitive to oedema and tissue degeneration [13]. Axial diffusivity (AD) refers to the diffusion parallel to the axon path (a decrease in AD can indicate axon damage, but myelin does not influence AD). Another parameter, radial diffusivity (RD), assesses the degree of diffusion perpendicular to the axons’ course. An increase in RD may indicate demyelination, axonal loss, or reduced axon packing density [13].

Magnetisation transfer imaging (MTI) and the magnetisation transfer ratio (MTR) are techniques that reveal structural alterations in tissue by detecting the differences between water molecules bound and unbound to macromolecules [14]. A lower MTR may suggest, for example, a lower myelin content in the brain tissue or the occurrence of neuroinflammation [15,16].

T2 relaxometry is an advanced MRI method that quantitatively measures the T2 relaxation time in a specific tissue. It can be useful in the detection of demyelination, as well as neurodegeneration and oedema [17].

T2*-weighted imaging measures the effective T2 relaxation, which is sensitive to magnetic field inhomogeneities. This sequence is used in imaging hemosiderin deposits, deoxygenated haemoglobin, or methaemoglobin and is useful in detecting bleeding and microbleeds [18]. q-T2* is a quantitative method for measuring effective T2 relaxation that can accurately assess the level of iron in tissues as well as indicate demyelinating lesions [19].

Susceptibility-weighted imaging (SWI) sequences offer significant advantages in detecting white matter hyperintensities and microhemorrhages in cases of vascular dementia and cerebral amyloid angiopathy. In addition, SWI has been shown to enhance the detection of certain findings that are not clearly visible on standard T2*-weighted images, like nigrosome 1 in Parkinson’s disease and dementia with Lewy bodies, as well as the central vein and peripheral rim signs in multiple sclerosis [8,20].

**Figure 1:**
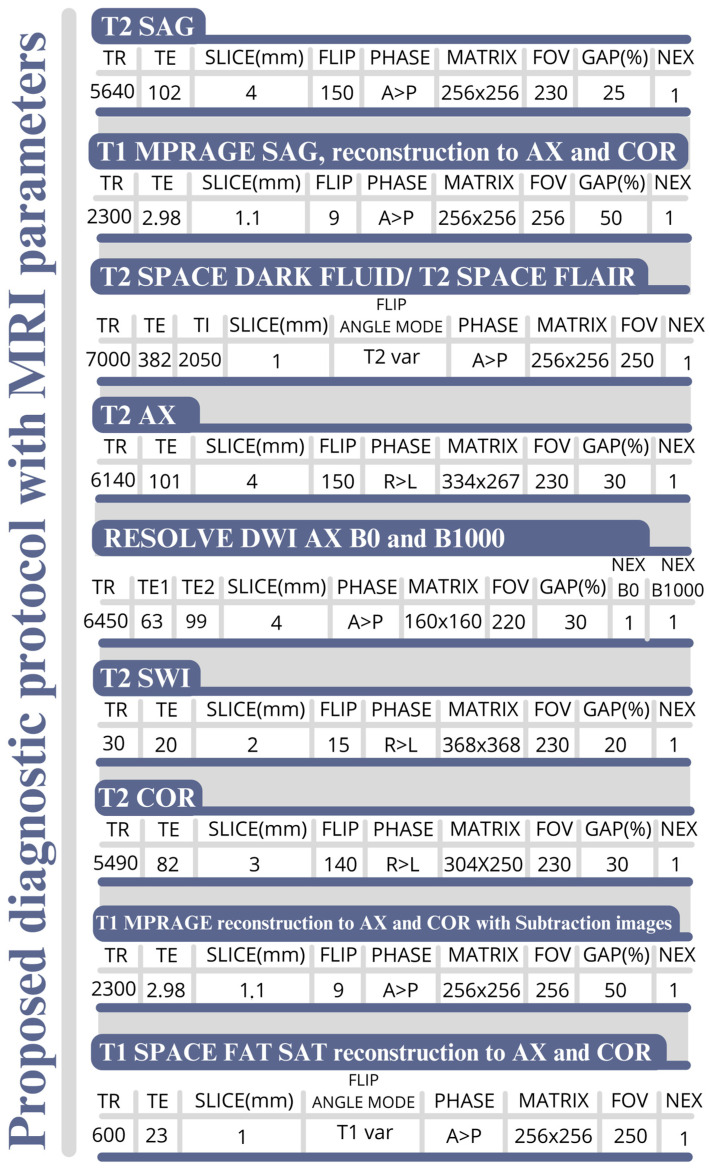
Proposed MRI protocol for demyelinating diseases and encephalitis—TOTAL SCAN TIME (3T MRI) = 26 min 20 s. (AX—axial, COR—coronal, DWI—diffusion-weighted imaging, FAT SAT—Fat Saturation, FLAIR—Fluid-Attenuated Inversion Recovery, FOV—field of view, MPRAGE—Magnetization Prepared Rapid Gradient Echo, NEX—number of excitation, RESOLVE—Readout SEgmentation Of Long Variable Echo-trains, SAG—sagittal, SPACE—sampling perfection with application-optimised contrasts using different flip-angle evolutions, SWI—Susceptibility-weighted imaging, TI—inversion time, TR—repetition time).

**Figure 2:**
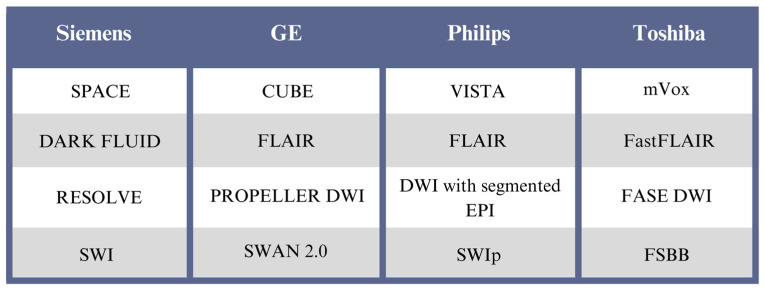
Sequence acronyms across the main MR vendors.

Based on some of the MRI sequences mentioned above, we would like to propose an MRI protocol for the neuroimaging of demyelination and neuroinflammation (Figure 1). To facilitate clinical work and navigation of scientific papers using MRI from different vendors, Figure 2 shows the acronyms for the MRI sequences used by the main manufacturers.

## 4. Inflammatory Demyelinating Diseases of the Central Nervous System

Inflammatory demyelinating diseases are a heterogeneous group of disorders with an acute or chronic inflammatory process underlying their aetiopathogenesis, which includes multiple sclerosis (MS), neuromyelitis optica (NMO), and neuromyelitis optica spectrum disorders (NMOSD), acute disseminated encephalomyelitis (ADEM), and myelin oligodendrocyte glycoprotein antibody-associated demyelination (MOGAD) [21]. To our knowledge, PET/MRI has primarily been used in MS research, but its potential is also indicated in ADEM.

### 4.1. Multiple Sclerosis

MS is the most common non-traumatic cause of disability among young adults [22], with an incompletely elucidated aetiology that takes into account complex interactions between genetic, environmental, and lifestyle factors [23]. Demyelination, central nervous system (CNS) inflammatory lesions, and axonal degeneration are observed in the course of the disease [24].

The International Advisory Committee on Clinical Trials of MS describes four types of MS, also known as the Lublin classification, including clinically isolated syndrome, relapsing-remitting MS, secondary progressive MS, and primary progressive MS [25,26].

Clinically isolated syndrome (CIS) is defined as the initial manifestation of neurological symptoms resulting from inflammation and demyelination within the central nervous system, with a duration of a minimum of 24 h and potential progression to MS. The predominant proportion of patients initially exhibit a relapsing-remitting course (RRMS), which is distinguished by the occurrence of relapses (flare-ups or attacks) of symptoms, subsequently followed by periods of partial or complete recovery (remissions) [27]. The onset of secondary progressive MS (SPMS) is invariably preceded by RRMS. The disease transitions to a stage in which there are fewer relapses and a more gradual progression of symptoms and disability. Primary progressive MS (PPMS) is characterised by a progressive decline in neurological function from the onset of symptoms, without early relapses or remissions, ordinarily diagnosed at a later stage in life [25,26,27,28,29].

MRI is the main diagnostic and monitoring tool for MS, which typically shows focal hypointense lesions on T1 sequences, while T2 and FLAIR sequences reveal hyperintense lesions [2,3]. The use of ultrahigh-field MRI and advanced MRI techniques (DTI, MTI, DWI, and T2 relaxometry) may indirectly indicate demyelination [2,16], but these methods are not specific for quantifying myelin changes, as they are influenced by other conditions such as the presence of inflammatory infiltrate, oedema, intracellular and extracellular water, or axonal damage [30]. A more specific method for quantifying myelin alterations is PET using radioligands that bind to myelin. In MS, PET can also be used to investigate the pathophysiology of CNS inflammation by utilising markers of microglial activation, as well as to assess neuronal dysfunction [31]. The combination of PET and MRI in a PET/MRI hybrid system can provide a number of benefits by simultaneously obtaining complementary anatomical and functional data from both modalities.

#### 4.1.1. Demyelination

Studies indicate that radiolabels such as [^11^C]-labelled Pittsburgh Compound-B ([^11^C]-PIB), [^18^F]florbetaben, and [^18^F]florbetapir, which are used in the quantification of amyloid-β among patients with Alzheimer’s disease [32], can also be used to quantify myelin content [33].

Studies using [^18^F]florbetapir or [^11^C]-PIB and PET/MRI found that lesions visualised in T1/T2 MRI sequences among MS patients had a lower distribution volume ratio (DVR) compared to the DVR of the normal-appearing white matter (NAWM) of MS patients, as well as the white matter (WM) of healthy subjects [30,34,35] However, Zhang et al. did not find this relationship in lesions smaller than 5 mm when comparing lesions in MS patients with NAWM [35]. Furthermore, the high intensity of T2 lesions was associated with a lower DVR, and parametric DVR maps were more sensitive in imaging demyelination within smaller T2 lesions than parametric SUV maps. The lesions detected were characterised by a centripetal decrease in radioligand binding, also involving the NAWM 2–8 mm around the lesions [30,34,35,36]. Nevertheless, research (except Pitombeira et al. [36]) has shown no differences between the averaged tracer uptake in the NAWM of MS patients and healthy subjects [34,35], which appears to be in opposition to the alterations detected outside of lesions using advanced MRI techniques. These alterations included a trend towards a lower mean FA and a higher RD (DTI parameters) in NAWM among MS patients, which could indicate micro demyelination involving the NAWM [35]. However, it is worth noting that MRI techniques are not specific to alterations in myelin integrity, and the results are also affected by factors such as axonal damage or microglia activation present in the NAWM [30]. Nevertheless, the use of hybrid PET/MRI with advanced MRI techniques has revealed a correlation between the reduced uptake of ^18^F-florbetapir in lesions visualised on the T1-w/T2-w sequences and an increase in MD, AD, and RD in these lesions. However, the correlation between FA and the uptake of this tracer in the lesions is unclear [34,35].

Some lesions visible in standard MRI sequences may show enhancement after the administration of a gadolinium(Gd)-based contrast agent [37]. Bodini et al. emphasise that Gd+ lesions were characterised by intermediate tracer uptake between the NAWM and the lesions observed in T2-weighted sequences, which may indicate that these lesions are affected by earlier stages of demyelination than T2-weighted MRI lesions [30].

The results of studies associating the degree of demyelination in specific CNS locations with clinical test outcomes are inconclusive. A study by Pitombeira et al. in 45 MS patients found that reduced DVR ^11^C-PIB values in the MRI lesions, corpus callosum, and caudatum were associated with greater patient disability (as measured by the Expanded Disability Status Scale (EDSS)) (Table 1) [36].

However, Carotenuto et al., in a study including 18 patients with RRMS, found that DVR [^18^F]florbetapir values in T1 and T2 lesions did not correlate with the clinical test scores (including EDSS) except the Timed 25-Foot Walk (25-FWT) [34] (Table 1). The authors suggest that an explanation for the inconsistency in results may be the greater precision of the 25-FWT test than the EDSS in detecting differences in patient disability. Conducted on 51 patients with MS, the Campanholo et al. study using hybrid PET/MRI and ^11^C-PIB to assess myelin imaging as a predictor of cognitive impairment and psychomotor speed revealed that data from advanced MRI techniques indirectly quantifying myelin in tissues–MTR (evaluated in the thalamus and corpus callosum) and DTI (FA) (in the thalamus and caudate) corresponded to the differences in patient’s cognitive status [38]. The association between FA and cognitive function coefficients was not as anticipated. Interestingly, patients with lower FA values, which indicate myelin loss, demonstrated better cognitive performance. However, the authors suggest that MTR, whose low values may indicate demyelination and increased inflammation [39], may serve as a good predictor of cognitive impairment. Only RRMS patients showed an association between ^11^C-PIB uptake and the MTR values in certain areas and cognitive function and psychomotor speed (Table 1) [38]. A meta-analysis by York et al. shows lower MTR levels in all brain regions of patients with RRMS compared to healthy individuals. Additionally, it confirms that these lower MTR values are associated with greater disability, as measured by the EDSS [40]. Therefore, MTR could be a valuable clinical parameter, highlighting its potential usefulness in future PET/MRI studies.

**Table 1:** Correlations between neuroinflammation and demyelination tracer uptake levels, derived from hybrid PET/MRI in specific brain structures, with symptoms of disability and cognitive impairment in patients with multiple sclerosis. (EDSS—Expanded Disability Status Scale; 9-HPT—Nine-Hole Peg Test; SDMT—Symbol Digit Modalities Test; CVLT-II—California Verbal Learning Test II; BVMT-R—Brief Visuospatial Memory Test-Revised; 25-FWT—25-Foot Walk Test; EF/IPS—Executive Functions/Information Processing Speed; PASAT—Paced Auditory Serial Addition Test; WLG—Word List Generation, TSPO—translocator protein).

	Scale	Radiotracer (Binding)	Quantitative Metric	Location of Tracer Uptake	Correlation	Research
	**neuroinflammation**					
**disability**	**EDSS**	^11^C-PBR28 **(TSPO)**	SUVR	cortex, thalamus, hippocampus, basal ganglia, NAWM, WM lesions	positive	Herranz et al. (2016) [41]
				cortical lesions		Herranz et al. (2020) [19]
				meningeal/parameningeal tissue		Herranz et al. (2024) [42]
				cerebellar lesions and NAWM, cNAGM		Barletta et al. (2020) [43]
		(R)-[^11^C]PK11195 **(TSPO)**	V_T_	cortical GM, cerebellar cortex, corpus callosum, caudatum, total T2-lesion, thalamus, NAWM		Pitombeira et al. (2022) [36]
	**9-HPT**	(R)-[^11^C]PK11195 **(TSPO)**	V_T_	cortical GM, cerebellar cortex, corpus callosum, caudatum, T2- lesions, NAWM	positive	Pitombeira et al. (2022) [36]
**cognitive impairment**	**SDMT**	^11^C-PBR28 **(TSPO)**	SUVR	thalamus, hippocampus, NAWM	negative	Herranz et al. (2016) [41]
				cortical lesions		Herranz et al. (2020) [19]
				cerebellar NAWM		Barletta et al. (2020) [43]
		(R)-[^11^C]PK11195 **(TSPO)**	V_T_	corpus callosum		Pitombeira et al. (2022) [36]
	**CVLT-II ** **BVMT-R**	^11^C-PBR28 **(TSPO)**	SUVR	temporal and occipital cortex, cingulate, prefrontal cortex, thalamus	negative	Herranz et al. (2016) [41]
	**demyelination**					
**disability**	**EDSS**	^11^C-PIB **(myelin,** **β -amyloid)**	DVR	corpus callosum, caudate, total T2-lesion	negative	Pitombeira et al. (2022) [36]
	**9-HPT**	^11^C-PIB **(myelin,** **β -amyloid)**	DVR	**RRMS group only** caudate, lesions, corpus callosum	negative	Campanholo et al. (2022) [38]
	**25-FWT**	[^18^F]florbetapir **(myelin,** **β -amyloid)**	DVR	**RRMS group** T1/T2 lesions	negative	Carotenuto et al. (2020) [34]
		^11^C-PIB **(myelin,** **β -amyloid)**	DVR	**RRMS group only** lesions	negative	Campanholo et al. (2022) [38]
**cognitive impairment**	**SDMT**	^11^C-PIB **(myelin,** **β -amyloid)**	DVR	corpus callosum	positive	Pitombeira et al. (2022) [36]
	**EF/IPS** **(SDMT+PASAT+WLG)**	^11^C-PIB **(myelin,** **β -amyloid)**	DVR	**RRMS group only** caudate, thalamus, corpus callosum, cortical GM, WM, NAWM	positive	Campanholo et al. (2022) [38]

Although Zhang et al. did not show a correlation between the uptake of [^18^F]florbetapir, FA, MD, AD, and RD in lesions and EDSS scores in the initial scan, they observed a reduction in disability (decrease in EDSS) in a follow-up scan of the same patients. Improvement in the EDSS results was associated with a decrease in the global demyelination index, as well as a decrease in areas of demyelination in damaged white matter (DWM) lesions demonstrated with [^18^F]florbetapir [35]. The authors suggest that a better reflection of clinical severity could be an assessment of remyelination or the balance between demyelination and remyelination rather than an assessment of demyelination only.

Du et al. indicated that radiomic data obtained with hybrid PET/MR using [^18^F]florbetapir may help to predict the annual relapse rate (ARR) among patients with RRMS [44]. Their proposed multi-modal model based on deep learning using radiomic data from PET/MRI predicted the risk of MS recurrence with greater accuracy than single-modality models based on PET or MR.

#### 4.1.2. Neuroinflammation

Herranz et al. conducted a series of studies on the neuroinflammatory process in MS using PET/MR (two of them also used 7T MRI) with the highly specific expression marker TSPO (translocator protein)-^11^C-PBR28 [19,41,42].

TSPO is an 18 kDa mitochondrial translocator protein expressed mainly in the outer mitochondrial membrane of steroid-synthesising cells in the central nervous system (microglia, astrocytes, endothelial cells, etc.) and peripheral tissues. TSPO participates in many physiological processes, including cholesterol transport into mitochondria, steroid hormone synthesis, and bioenergetics [45]. Positron emission tomography targeting of TSPO provides a molecular-specific approach to image and quantify the in vivo innate immune cell density in the central nervous system of the human brain, with a significant contribution of activated cells, including pro-inflammatory microglia. Activated microglia are the source of TSPO signalling, suggesting a link between chronic inflammation and neurodegeneration in multiple sclerosis [46].

Patients with MS were reported to have a higher uptake of the radiolabel in the whole brain, especially in the cortex and cortical lesions, as well as in the deep grey matter (GM) and NAWM, compared to healthy subjects [19,41,42]. In patients with secondary progressive multiple sclerosis (SPMS), higher whole-brain uptake of ^11^C-PBR28 has been observed compared to those with RRMS. In SPMS, the frontal and parietal areas appeared to be the main areas most severely affected by neuroinflammation, while in RRMS, the occipital and temporal regions showed greater involvement [41]. Another study revealed increased neuroinflammation in cortical lesions (visualised on T2*-weighted 7T MRI sequences) in both forms of MS, while abnormally high TSPO uptake in the normal-appearing cortex was found only in patients with SPMS, which may suggest CNS inflammation and disease progression [19]. There was also a higher percentage of active lesions in SPMS (62%) than in RRMS (42%). Differences in the two forms of the disease were also observed by analysing the correlations between q-T2* (whose increase is associated with greater demyelination or iron loss) and ^11^C-PBR SUVR (standardised uptake value ratio) in cortical lesions. In RRMS, a positive correlation was observed much more frequently. In contrast, in SPMS, the surface area of negatively correlated areas was larger, which the authors suggest could be related to inactive areas of chronic demyelination or tissue regeneration. The intensity of microglia activation in WM lesions in MS was relatively low. The reduction in cortical volume correlated with the severity of the neuroinflammatory response in the thalamus, which may suggest the spread of cortical pathology to the thalamus or the opposite.

The most recent of the studies, conducted on 49 MS patients, found that, in addition to the cortex, increased TSPO uptake was also present in the meninges (the SUVR ^11^C-PBR measured approximately 3 mm above the surface of the pia mater), indicating a role for meningitis in MS pathogenesis [42]. Interestingly, patients treated with second-line disease-modifying drugs did not show an increase in the TSPO signal in the meningeal and parameningeal tissue, which occurred in patients treated with first-line drugs. The classification of disease-modifying therapies (DMT) into first and second-line is becoming outdated. Instead, the terms moderate-efficacy DMT (meDMT) and high-efficacy DMT (heDMT) are now more commonly used. In this context, meDMT refers to first-line treatment, while heDMT corresponds to second-line treatment. The advantage of meDMT is that it has a low risk of side effects, although it is moderately effective in treating multiple sclerosis (MS). In contrast, heDMT has a higher risk of side effects but offers greater effectiveness compared to meDMT, which may explain the results obtained in the study [47]. The authors indicate that the severity of neuroinflammation examined by hybrid PET/MR is related to the clinical symptoms of MS patients (Table 1) [42].

Barletta et al., using the same TSPO expression marker (^11^C-PBR28) in PET-MRI and employing 7T MRI, demonstrated increased TSPO expression also in the cerebellum of MS patients [43]. Microglia activation, as measured by ^11^C-PBR28 SUVR, was found to be higher in cerebellar lesions compared to the whole cerebellum of healthy subjects, as well as the SUVR of the NAWM and cerebellar normal-appearing grey matter (cNAGM) of MS patients (even after exclusion of the perilesional area) was higher than in the WM and cortical GM, respectively, of the controls. Interestingly, the presence or absence of treatment did not affect microglia activation. The intensity of microglia activation in the cerebellum was also shown to be statistically significantly associated with the degree of disability and cognitive impairment of patients (Table 1).

Pitombeira et al., using PET/MR and two radiolabels: [^11^C]-PIB and the marker of innate immune cell activation-(R)-[^11^C]PK11195 (TSPO) in opposition to previous studies, found no significant difference in the distribution volume (VT) of (R)-[^11^C]PK11195 in any of the VOIs (Volume of Interest) tested between the MS patient group and the healthy subject group [36]. However, it should be noted that the first-generation TSPO tracer used is a lower sensitivity and specificity tracer than ^11^C-PBR28, and the study used a volume-based analysis with predefined anatomical regions, which may have influenced the study results. However, the authors found a diffusely increased uptake of TSPO in WM in patients with SPMS compared to healthy subjects and a lower uptake of TSPO in some regions of WM in patients with RRMS compared to the healthy group. This study supports the correlation of the severity of neuroinflammation with the severity of clinical symptoms (Table 1). Due to the observed lack of correlation between demyelination and innate immune cell activity in the anatomical regions studied, the authors suggest that these processes may be independent, which may account for the different disability profiles observed in patients with MS.

#### 4.1.3. Summary

In conclusion, hybrid PET/MRI systems, thanks to the improved contrast between normal and damaged brain tissue, the possibility to better identify areas affected by neuroinflammation and demyelination, as well as the simultaneous provision of radiomic data from the two imaging modalities, is a promising neuroimaging technique for patients suffering from MS. PET/MRI may help relate patients’ disabilities and cognitive deficits, to specific regions of CNS neuroinflammation and demyelination, as well as in predicting MS relapses or in assessing treatment efficacy.

The severity of neuroinflammation imaged by PET/MRI within lesions detected on MRI in the cortex, white matter, and cerebellum, as well as in the whole cortex and cerebellum, NAWM, limbic system structures, and meningeal tissue, may correlate with symptoms of cognitive impairment and severity of disability in patients with MS. Highlighting the inconclusive nature of the studies published to date, we indicate that these findings suggest that, particularly in RRMS patients, a lower uptake of myelin-binding radioligand in T1- and T2-weighted MRI lesions, the corpus callosum, and caudate may be associated with increased motor impairment. A correlation between cognitive impairment and increased demyelination in addition to these areas may also be observed in the thalamus, white matter, and cortex.

However, further longitudinal studies on larger groups of patients are required, which, in addition to the processes of neuroinflammation and demyelination, will also take into account the remyelination process. The direction of further research should also include evaluating the efficacy of MS treatment with PET/MRI (including an assessment of the severity of the inflammatory process involving the meninges). It may also be helpful to use two radioligands (both a marker of demyelination and neuroinflammation—preferably second-generation TSPO) in the same patients to provide more insight into the pathogenesis of MS and to clarify the connections between these processes. We also suggest that advanced MRI techniques (MTR, DTI) be included in further PET/MRI studies of demyelination and neuroinflammation due to the correlation of these parameters with the severity of clinical symptoms, as well as the severity of demyelination and inflammation.

### 4.2. Acute Disseminated Encephalomyelitis

ADEM is an acute demyelinating inflammatory CNS disease involving multifocal areas of the WM, less commonly the GM and spinal cord, which typically shows a temporal association with infectious disease or vaccination [48,49].

Zhang et al. first described the use of hybrid PET/MRI using ^18^F-florbetapir in imaging demyelination in a patient with ADEM [50]. The authors observed a significantly lower SUVR within the multifocal hyperintense lesions visualised on T2 FLAIR than on NAWM, indicating demyelination of these areas, which supported an accurate diagnosis of ADEM. PET/MRI was also used to assess the response to treatment, visualising a reduction of lesions’ hyperintensity and an increase in SUVR within the DWM, but this was still lower than normal, suggesting incomplete remyelination.

Lehaus et al. pointed out that PET/MRI using ^18^F-fluoro-ethyl-tyrosine (^18^F-FET), which is mainly used in neurooncology, was helpful in the diagnosis of ADEM in a patient with MRI findings suggestive of brain lymphoma [51]. Within the most extensive hyperintense lesion visible on MRI, heterogeneous ^18^F-FET uptake was observed, while perivascular lesions showed low tracer uptake. The ADEM diagnosis was confirmed by biopsy and histopathological examination.

The above clinical case reports suggest that PET/MRI may be a useful diagnostic tool in ADEM to assist in the differentiation of ADEM from other disease entities, as well as to help assess the efficacy of treatment. However, further studies on larger groups of patients are necessary to evaluate the real benefit of using this method among patients with ADEM.

## 5. Encephalitis

Encephalitis is most often caused by a virus, with Herpes Simplex Virus (HSV) being the most common. However, our review of the literature indicates that studies using PET/MRI have primarily focused on autoimmune encephalitis, which has become increasingly recognised [52].

### 5.1. Autoimmune Encephalitis

AIE refers to a heterogeneous group of central nervous system disorders in which the immune system generates autoantibodies that target neuronal surface antigens, synaptic receptors, and intracellular proteins. AIE presents with a wide spectrum of neurological manifestations, including cognitive impairment, motor dysfunction, psychiatric symptoms, epilepsy, and other potentially irreversible sequelae [53,54]. In addition to blood serum and cerebrospinal fluid (CSF) analyses, as well as electroencephalography (EEG) and a clinical examination, neuroimaging plays an essential role in diagnosing and guiding therapeutic decisions in cases of known or suspected autoimmune encephalitis [53,55]. The most commonly employed imaging technique is brain MRI. While MRI is the initial and most frequently utilised imaging modality within the AIE patient demographic, it may manifest as normal or unremarkable in select patients [56]. Compared to traditional MRI, PET-MR is perceived to exhibit enhanced sensitivity in evaluating intracranial lesions. The concurrent utilisation of both methodologies can facilitate more expedient and precise diagnostic determinations [57].

Zhang et al., in their study using hybrid PET/MR, compared the diagnostic utility of ^18^F-DPA-714 PET with conventional MRI in 25 AIE patients and assessed the correlation between TSPO PET uptake and clinical features [53]. The study revealed a positive detection rate of 72% for AIE when using ^18^F-DPA-714 PET, as opposed to 44% for conventional MRI. Despite these figures, the statistical significance was not established. Among patients who showed alterations on PET as well as MRI, a greater range of abnormalities was often observed on positron emission tomography than on MRI, while in two patients, MRI showed lesions with a normal PET scan. Additionally, patients who experienced seizures demonstrated significantly elevated mean SUVR in the cerebral cortex than patients without seizures. In the group of 13 patients who underwent follow-up PET/MRI scans, 85% exhibited a reduction in ^18^F-DPA-714 uptake, which co-occurred with an improvement in symptoms following immunosuppressive therapy. Similar observations were also described by Meng et al., who, using ^18^F-DPA714 PET/MR in patients with seronegative AIE, found that 10/15 (67%) patients showed lesions on PET, while only 3/15 (20%) patients revealed lesions on MRI [58]. In contrast, four patients (27%) did not show lesions on either technique. The authors also found that an increased tracer uptake correlated with the Clinical Assessment Scale for Autoimmune Encephalitis (CASE) score. Furthermore, the presence of ataxia was associated with a significantly higher SUVR in the cerebellum than in patients without ataxia. In 50% (5/10) of patients, a decrease in tracer uptake was found at follow-up. These findings indicate the potential usefulness of ^18^F-DPA-714 PET in conjunction with MRI in the diagnosis of AIE (due to the complementary results of the two methods) and in the control of treatment efficacy. However, we would like to point out that ^18^F-DPA-714 is a marker that detects microglial activation during neuroinflammation but is not specific to AIE. Therefore, while PET/MRI with this tracer can provide valuable diagnostic insights, it is essential to consider the patient’s symptoms, results from other tests, and the appropriate diagnostic criteria when diagnosing AIE [59].

Deuschl et al. studied the use of ^18^F- fluorodeoxyglucose (^18^F- FDG)-PET/MRI for diagnosing limbic encephalitis. Hybrid imaging detected abnormalities in 95% of patients (19/20), demonstrating superior sensitivity compared to single imaging modalities: MRI at 80% (16/20) and PET at 50% (10/20) [60]. Gallus et al. also highlight the diagnostic potential of [^18^F]DPA-714 PET-MRI in autoimmune limbic encephalitis [61]. The authors found that increased uptake of the microglial activation tracer occurred asymmetrically in both mesial temporal lobes, with the lateralisation of increased uptake corresponding to mesial temporal lobe lesions on FLAIR-MRI and abnormalities on the anterior temporal EEG.

A further study demonstrated the efficacy of FDG PET/MRI and PET/CT (computed tomography) in supporting the diagnosis of AIE among children, as well [57]. Aydos et al. [57] conducted a retrospective analysis involving six seronegative paediatric patients with a preliminary diagnosis of autoimmune encephalitis, only two of whom (33%) presented abnormalities on a previous MRI scan. The initial FDG PET and statistical parametric mapping method analysis findings were abnormal in all patients, with four cases exhibiting only hypometabolism. All patients had metabolic abnormalities in the temporal lobes. Additionally, visual and semiquantitative FDG PET findings revealed hypometabolism in extratemporal regions. One patient exhibited a hypermetabolic pattern in the right mesiotemporal lobe, while another patient demonstrated a mixed hypohypermetabolic pattern, characterised by hypermetabolism in the left mesial temporal lobe, and hypometabolism in the left frontal, parietal lobes, posterior cingulate gyrus, and occipital lobe. Interestingly, the areas affected on the MRI scan (mesial temporal lobes) corresponded to areas of hypermetabolism on PET imaging. It is worth noting that the alterations in brain tissue metabolism detected in the FDG PET/MRI scan may indicate an inflammatory process occurring in the course of AIE but are in no way specific to this disease entity. When diagnosing a patient, it is important to remember to correlate the results of the PET/MRI scan with clinical indicators and the results of other tests.

Simultaneous (^18^F-FDG) PET/MRI was the sole factor facilitating the advancement toward a diagnosis of seronegative AIE in the case described by Taneja et al. [62]. PET/MRI revealed an increased uptake of radiotracer in both temporal lobes and basal nuclei (caudate nuclei and putamen), while the MRI component revealed a mild FLAIR and T2 hyperintensity affecting the bilateral medial temporal lobes, including the hippocampus, as well as the bilateral basal ganglia and cingulate gyrus with minimal gyral thickening/swelling.

Other cases of AIE have been reported in the literature in which PET/MRI using ^18^F-FDG and ^18^F-DPA-714 played a key role in diagnosis. The authors indicate that the use of hybrid PET/MRI can increase diagnostic confidence in seronegative AIE cases [63]. They also describe patients with anti-NMDAR [64], voltage-gated potassium channel antibody [65], anti-Ma1 and anti-Ma2 [66], anti-LGI1 [67,68], anti-CASPR2 [67,69], against SOX1 [69], anti-GABA A receptor [70], and anti-DPPX [71].

In conclusion, employing a combined PET/MRI approach with quantitative analysis in cases of potential autoimmune encephalitis may be a very effective method to support diagnosis. Simultaneous PET/MRI offers an advantage over single-modality imaging techniques by combining high-resolution anatomic and functional information from MRI with metabolic information from PET within the same imaging session. The metabolic information derived from ^18^F-FDG or ^18^F-DPA-71 PET has been demonstrated to be particularly beneficial in patients with inconclusive or negative MRI results. The use of PET/MRI may also help to accelerate the diagnosis of AIE in seronegative patients. The observed abnormalities may also reflect clinical symptoms in patients with AIE and may prove useful in monitoring the course of the disease.

### 5.2. Viral Encephalitis

There is a shortage of studies and clinical case reports exploring the use of hybrid PET/MRI in viral encephalitis. To the best of our knowledge, the application of PET/MRI has been documented for viral encephalitis caused by HSV and Human Immunodeficiency Virus (HIV).

Schillaci et al., using special software, obtained ^18^F-FDG PET/MRI images by overlaying images from separately acquired PET and MRI scans of a patient with Herpes Simplex Encephalitis (HSE) [72]. In addition to extensive areas of hypometabolism in the left temporal, parietal, and occipital lobes coinciding with areas of hyperintensities in T1- and T2-weighted images, a decreased uptake of radiolabel in the left thalamus and striatum was demonstrated, which did not correspond with any alterations on MRI. A focal increased ^18^F-FDG uptake was also observed in the inferior left temporal cortex, corresponding to a focal area of contrast enhancement in T1-weighted scans, which was described as a focal inflammatory process with meningeal vasodilation, indicating active viral replication. The authors believe that the PET/MR images obtained may support the identification of active foci of inflammation, as well as the assessment of the prognosis and treatment of patients with HSE.

According to Pompanin et al., valuable data in terms of diagnosis, association of functional brain alterations with clinical symptoms, as well as assessment of treatment response and prognosis ^18^F-FDG PET/MRI can also be provided in patients with HIV encephalitis [73]. The case study refers to a patient with HIV encephalitis who developed opsoclonus-myoclonus syndrome (OMS). OMS is a rare neurological syndrome clinically characterised by either opsoclonus (rapid, repetitive, multidirectional eye movements that persist during sleep) together with myoclonus or the triad of opsoclonus, myoclonus, and ataxia [74]. Aetiology usually includes paraneoplastic entities and infectious, metabolic, or idiopathic causes. The pathophysiology is uncertain, and its neuroanatomical correlation is debated, but it usually implies lesions in the cerebellum, midbrain, thalamus, and paramedian pontine reticular formation [74,75,76]. In a patient with OMS, the study showed extensive cortical hypometabolism (with sparing of the sensory-motor cortex bilaterally) and increased glucose uptake in the cerebellum, colliculus, red nucleus, and substance nigra, explaining the patient’s symptoms [73]. It is suggested that hyperactivation of the dentato-rubral pathway may occur in the course of OMS, which corresponds to the hypermetabolism in the red nucleus observed in the study. Research using PET indicates that subcortical hypermetabolism and cortical hypometabolism are associated with the presence of opsoclonus [77]. In contrast, alterations in cerebellar metabolism may correlate with the patient’s ataxia [78]. At follow-up 4 months later, there was a decrease in glucose uptake in areas of hypermetabolism and an increase in uptake in previously demonstrated regions of hypometabolism, which corresponded with clinical improvement [73].

### 5.3. Parasitic Encephalitis

There is a lack of work describing the use of hybrid PET/MRI in encephalitis of parasitic aetiology. However, based on the results of the independent use of ^18^F-FDG PET/CT and MRI in neurocysticercosis, we suggest that hybrid PET/MRI is a method worth investigating, as it could have potential benefits (i.e., facilitating the diagnosis and differentiation of parasitic encephalitis, in which MRI findings alone may be atypical and suggest other diagnoses) [79].

## 6. Conclusions

PET/MRI utilising relevant radioligands to image the severity of demyelination and inflammation, as well as indicate metabolic alterations within the brain, holds great potential for assisting clinicians in their practice. In the context of inflammatory demyelination, PET/MRI is valuable for monitoring disease progression, clarifying symptoms related to cognitive impairment, and evaluating the severity of disability in patients. Additionally, it may help assess treatment effectiveness and predict relapses in patients with MS. In cases of encephalitis, especially AIE, studies have demonstrated that PET/MRI is superior to other imaging modalities, particularly in terms of diagnosis and differentiation. This technique also has the potential to identify active foci of inflammation, monitor the progression of the disease, and evaluate responses to treatment. To fully evaluate the potential of hybrid PET/MRI, further longitudinal studies are necessary involving larger groups of patients with inflammatory demyelinating diseases. These studies should encompass a range of conditions within this category, including ADEM, NMO, NMOSD, and MOGAD, in addition to multiple sclerosis. Additionally, more studies using PET/MRI in the field of encephalitis are needed, which should include research on viral and parasitic encephalitis in addition to the AIE studied so far.

## Data Availability

The data supporting the findings of this study are available within the article.
